# Regulatory Framework of Fortified Foods and Dietary Supplements for Athletes: An Interpretive Approach

**DOI:** 10.3390/nu13113858

**Published:** 2021-10-28

**Authors:** Susana Menal-Puey, Iva Marques-Lopes

**Affiliations:** AgriFood Institute of Aragon (IA2), Faculty of Health and Sport Sciences, University of Zaragoza, 22002 Huesca, Spain; imarques@unizar.es

**Keywords:** regulatory framework, sport foods, fortified foods, dietary supplements, labels, informed choices, sport nutrition claims, sport health properties

## Abstract

A varied and well-planned diet can meet the nutritional needs of an athlete; however, in certain cases, it could be advisable to increase the intake of some vitamins, minerals or other components through the controlled intake of fortified foods or dietary supplements. In the European Union, a high number of sport foods and supplements are marketed; athletes could at times consume them indiscriminately or even choose products that have not been evaluated and approved by scientific evidence. In this sense, it is necessary to know and interpret the specific regulations for these products in order to make adequate use of them. The aim of this manuscript is to describe the current status of the European regulatory framework, focusing on: (1) regulation of the marketing and labelling of both fortified foods and supplements; (2) regulation of the use of substances used as ingredients in fortified foods; and (3) regulation of nutritional claims and/or health properties associated with nutrients, ingredients and other related substances. This review can facilitate knowledgeable decision making by sports nutrition professionals in order to counsel or manage adequate food choices as well as help consumers make better-informed food decisions. Other experts, such as producers who ensure food safety, might also be interested in this review.

## 1. Introduction

An adequate diet can provide all the nutrients necessary for the development and maintenance of the organism; however, in specific situations, such as the sport field, it may be necessary to increase the intake of some of these nutrients through the use of fortified foods and/or dietary supplements.

Fortified foods are common foods with one or more of their nutritional components increased in relation to their usual composition [1]; however, food supplements are not complete foods consisting instead of concentrated sources of nutrients or other substances with a specific nutritional or physiological effect, and they are marketed in dosed form (capsules, pills, tablets, powder sachets, liquid ampoules, dropper bottles or powders) to be taken in small unit quantities [2].

Although is supposed that a well-balanced diet could meet the increased energy and nutrient demands of athletes, the use of fortified foods or supplements may be necessary depending on the intensity/duration of the exercise or when food intake is not possible for various reasons [3]. In recent years, the intake of these products has grown [4] and dietary supplementation has become a common strategy to achieve a specific health status or performance benefit [5].

Appropriate use of these products can benefit athletes, but indiscriminate use could harm their health and performance [6]. In addition, there are risks associated with the intake of dietary supplements, related to the presence of harmful substances or even doping agents [7]. It is critical to provide information about the patterns of use and purchase of these products in athletes, in order to protect consumers from improper use and ensure informed choices; thus, a regulatory framework has been developed.

The present study explores and summarizes the legislative documents that regulate the marketing and labelling of fortified foods and dietary supplements for athletes in the European framework. The evolution of the regulatory framework is described, the mandatory information including in labelling is presented, and the legal use of vitamins, minerals and other chemical constituents in foods and supplements is discussed. This paper also outlines requirements for labelling, notification, and for claims of nutritional and/or health properties associated with a nutrient, ingredient, or any other substance.

This review provides knowledge to sport nutrition professionals in order to counsel or manage adequate food choices, and provides a nutritional education approach that will reduce the risk associated with nutritional supplementation and allow for making better-informed food decisions. It includes examples of some labels as an interpretative education approach.

## 2. Materials and Methods

Firstly, the topic “foodstuffs for particular nutritional uses” was taken into consideration, in order to understand the possible existence of a particular legal policy for sport foods.

Then, a narrative review of legislative documents related to fortified foods and dietary supplements was developed. The information was extracted from legislative documents such as laws, regulations, and/or standards laid down by the competent authorities in the European or in some cases national field.

The legislative documents selected for this review were those that included information about the different points established in the objectives of this review: (1) Regulation of the marketing and labelling of both fortified foods and supplements; (2) Use of substances as ingredients for food fortification; (3) Use of nutritional claims and/or claims of health properties associated with a nutrient, ingredient or any other substance.

The section “labelling and nutrition” on the European Commission website was consulted (https://ec.europa.eu/food/safety_en: accessed on 5 July 2021). This section compiles the European laws that establish the rights of consumers to safe food and to accurate and up to date information. 

According to the first objective of this review (marketing and labelling of both fortified foods and supplements) the subsection “food information to consumers” of the section “labelling and nutrition” was examined. 

In order to describe the correct use of substances as ingredients for food fortification (objective 2), the subsection “addition of vitamins and minerals” was studied. This regulatory section considers a wide range of nutrients such as vitamins, minerals, amino acids, essential fatty acids or fiber added to food in order to enrich or fortify them. 

In addition, the subsection “food supplements” was examined in order to explorer the harmonized list of vitamins and minerals or other substances that may be added for nutritional purposes in food supplements.

Finally, the subsection “nutrition and health claims” was examined to summarize the regulatory framework that controls the use of nutritional claims and/or claims of health properties associated with a nutrient, ingredient or any other substance in sport foods and dietary supplements. This broad group of rules is the legal framework used by food business operators when they want to highlight the particular beneficial effects of a product in relation to health and nutrition on the product label or in its advertising.

## 3. Results and Discussion

### 3.1. Topic “Foodstuffs for Particular Nutritional Uses”

In 1989, Directive 89/398/EEC of the European Council [8] regulated the topic “foodstuffs for particular nutritional uses”, including within this group the foods adapted to intense muscle wasting, especially for athletes. This regulation contemplated marketing and labelling standards for sport foods, but left open the possibility of establishing specific provisions applicable to certain groups of foodstuffs by means of specific Directives.

For years, many countries were in favor of regulating sport foods in a specific way; however, the European Commission did not consider it necessary to establish specific regulations. In 2009, the Directive of 1989 was derogated by Directive 2009/39/EC of the European Parliament and of the Council [9], which regulated foods for athletes and maintained the possibility of adopting specific provisions for foods adapted to intense muscle wasting. In 2013, these provisions were not established, and the new regulation, Regulation 609/2013 of the European Parliament and of the Council [10], left sport foods out of the particular foodstuffs and urged the European Commission to rule on this issue. 

Finally, in 2016, The European Commission decided that specific provisions were not necessary, establishing that these foods (including food supplements) should be treated as “ordinary foods” and regulated by horizontal European regulations.

### 3.2. Regulation of the Marketing and Labelling of Fortified Foods and Supplements

As indicated in the previous section, packaged foods for athletes, fortified or not, are regulated by the same horizontal regulations as common foods. In this way, the marketing and labelling of food for athletes is regulated by Regulation 1169/2011 of the European Parliament and of the Council [11], the legislative document related to food labelling, presentation and advertising of foodstuffs in the European Union. This framework provides the food information on the labelling of packaged foods, guarantees a high level of consumer protection and enable consumers to make informed decisions, preventing misleading actions and omissions of information.

According to this Regulation, all the sport foods must include the follow specific information:

(a) Name of the food
(b) List of ingredients
(c) Quantity of certain ingredients or category of ingredients
(d) Presence of substances or products causing allergies or intolerances
(e) Weight or net volume
(f) Minimum durability date, ‘use by’ date and date of freezing
(g) Special storage conditions and/or conditions of use
(h) Name and address of manufacturer
(i) Country of origin or place or provenance
(j) Instructions for use where it would be difficult to make an appropriate use
(k) Nutritional information

In addition to this information, the labels on dietary supplements should include other information regulated by Directive 2002/46/EC of the European Parliament and of the Council [12]. This regulation referred to the following particulars requirements for dietary supplements:

(a) The names of the categories of nutrients or substances that characterize the product or an indication of the nature of those nutrients or substances
(b) The portion of the product recommended for daily consumption
(c) A warning not to exceed the stated recommended daily dose
(d) A statement about that food supplements should not be used as a substitute for a varied diet
(e) A warning about that the products should be stored out of the reach of young children

Table 1 presents all the mandatory requirements for all sports in foods, fortified or not, with a brief description of each one. Athletes should consult this information in order to make better-informed choices.

All the information expressed in Table 1 must be printed on the package or on the label in such a way as to ensure clear legibility, in characters using a minimum font size where the x-height is equal to or greater than 1.2 mm, or, in case of containers with an area of less than 80 cm^2^, the x-height could be equal to or greater than 0.9 mm.

Regarding nutrition information and in order to share the same international approach on nutrition labeling, the European Regulation is in accordance with the Codex Alimentarius Guidelines on nutrition labelling [13], The Codex Alimentarius is a joint FAO/WHO food standards, guidelines and codes of practice adopted by the Codex Alimentarius Commission to protect consumer health and promote fair practices in food trade. It is important to note that these guidelines could be a supporting document to increase the understanding of consumer on nutrition labeling of food.

### 3.3. Use of Substances as Ingredients in Food Fortification

A wide variety of nutrients and other ingredients such as vitamins and minerals, amino acids, essential fatty acids, fibers or various plants and herbal extracts can be used to manufacture fortified foods and dietary supplements, as long as they have been approved by the European Food Safety Authority.

In this sense, a legislative framework that regulates the use of different substances as ingredients has been developed in order to establish requirements to ensure their safe use in foods.

For years Member States have developed different national regulations that obstructed the internal market; subsequently, harmonized rules were adopted, but only for the use of vitamins and minerals as ingredients. In 2002, Directive 2002/46/CE of the European Parliament and of the Council [12] was developed to regulate the use of vitamins and minerals in food supplements. Four years later, in 2006, vitamins and minerals in fortified foods were added by Regulation 1925/2006 of the European Parliament and of the Council [14], thus controlling both dietary supplements and fortified foods.

The list of authorized vitamins and minerals as ingredients included in these regulations has been reviewed, and they have been updated on several occasions. The last positive list of vitamins, minerals and their forms to add to food and supplements was compiled in Regulation 1170/2009 of the Council [15], amending Directive 2002/46/EC and Regulation (EC) No 1925/2006, as previously mentioned. It is important to note that fortified foods or food supplements containing vitamins and/or minerals or sources of them, which are not authorized under this Regulation framework, are prohibited, and should not be used by consumers.

In addition, consumers should know that fortified foods and food supplement labels must include information about how the product contributes to the daily diet. In this sense, they are required to include information about the percentage of the recommended daily amount of each nutrient that is met per 100 g or 100 mL, in a mandatory way. Optionally, information per individual packaged food or per dietary supplement can be added. The daily reference intakes of vitamins and minerals have been set by European legislation about food information to consumers, as mentioned in Table 1 [11].

Although vitamins and minerals have been regulated in a European context, the use of other substances in the manufacture of foods and supplements may be regulated by national rules in each Member State, and the European Commission, on the basis of the information provided by each Member States, may include them in the list of substances whose use in foods is prohibited, restricted or under scrutiny. In this sense, and on the basis of the assessment of a Member State (Germany), there is a harmonized negative list of substances with possible harmful effects. Commission Regulation (EU) 2015/403 [16] and Commission Regulation (EU) 2019/650 [17], incorporating, as prohibited substances, *Ephedra herbs* and its preparations from the genus *Ephedra* and *Yohimbe bark* and its preparations from *Yohimbe* (*Pausinystalia yohimbe*), respectively.

With respect to positive substances different from vitamins and minerals, there are not harmonized lists in a European context; however, several Member States have drawn up lists of substances that can be used in the production of food supplements. For example, Spanish Royal Decree 130/2018 [18] expanded the lists of vitamins and minerals, allowing eight categories of compounds in the manufacture of food supplements:

(a) Fatty acids
(b) Amino acids (and their salts of Na, K, Ca, Mg and HCl) and other nitrogenous compounds
(c) Dipeptides and peptides
(d) Coenzymes
(e) Flavonoids, carotenoids
(f) Nucleotides
(g) Polysaccharides and oligosaccharides
(h) Other substances

In this regard, it is usual to send food supplements from other Member States in a Member State with more lax regulations regarding authorized ingredients, by means of the mutual recognition principle. This principle was set out in Regulation (EC) 764/2008, of the European Parliament and of the Council [19], and established that a Member State cannot prohibit the sale, within its territory, of products legally marketed in another Member State, even if they have been manufactured in accordance with different regulations.

It is important to emphasize that, the lack of a harmonized European framework in terms of substances allowed in food supplements (other than vitamins and minerals) makes possible the commercialization of food supplements with ingredients other than those allowed in a Member State, through the principle of mutual recognition. Nevertheless, no Member State may market foods with the following prohibited ingredients: *Ephedra herbs* and its preparations from the genus *Ephedra* and *Yohimbe* bark and its preparations from *Yohimbe* (*Pausinystalia yohimbe*).

### 3.4. Nutritional Claims and/or Health Properties Associated with a Nutrient, an Ingredient or Any Other Substance

Finally, it should be noted that both, fortified foods and dietary supplements, may contain nutrition and/or health claims associated with a nutrient, ingredient or any other substance on the label. Regulation (EC) No 1924/2006 of the European Parliament and of the Council [20] lays down specific provisions about the use of nutrition and health claims concerning fortified foods or dietary supplements to be delivered as such to the consumer.

A nutritional claim means any claim which states, suggests or implies that a food has particular beneficial nutritional properties due to (a) the energy it provides, or provides at a reduced or increased rate, or does not provide; (b) the nutrients or other substances it contains, or contains in reduced or increased proportions, or does not contain. A health claim means any claim that states, suggests or implies that a relationship exists between a food category, a food or one of its constituents and health.

The nutrition or health claims must be authorized and subject to certain conditions of use and could refer to substances of mandatory declaration or others that do not appear on the list of mandatory mentions, in which case, the quantity of that substance must be indicated in the same visual field as the nutritional labelling. 

The allowed nutritional claims are listed in the annex of the aforementioned Regulation. Consumers should be aware that only the following mentions may appear on the labels; any other are not authorized: low energy, energy-reduced, energy-free, low-fat, fat-free, low-saturated fat, saturated fat-free, low sugar, sugar-free, with no added sugar, low sodium/salt, very low sodium/salt, sodium-free or salt-free, source of fibre, high fibre, source of protein, high protein, source of (name of vitamin/s) and/or (name of mineral/s), high (name of vitamin/s) and/or (name of mineral/s), contains (name of the nutrient or other substance), increased (name of the nutrient), reduced (name of the nutrient), light/lite, naturally/natural.

The health claims shall only be allowed if they are authorized in accordance with the Regulation and if they are included in later lists of claims. Table 2 includes those that have been authorized for commercial use. 

Athletes should know that, when a product is associated to a health claim, the label should include the following information:

(a) A statement indicating the importance of a varied and balanced diet and a healthy lifestyle.
(b) The quantity of the food and pattern of consumption required to obtain the claimed beneficial effect.
(c) Where appropriate, a statement addressed to persons who should avoid using the food.
(d) An appropriate warning for products that are likely to present a health risk if consumed to excess.

In order to share the same international approach on nutritional claims and health properties, the European Regulation takes into account the Codex Alimentarius guidelines for use of nutritional and health claims [21]. Consumers and experts can consult these guidelines to increase their understanding.

## 4. An Interpretive Approach to the Legislative Framework

**Figure 1:**
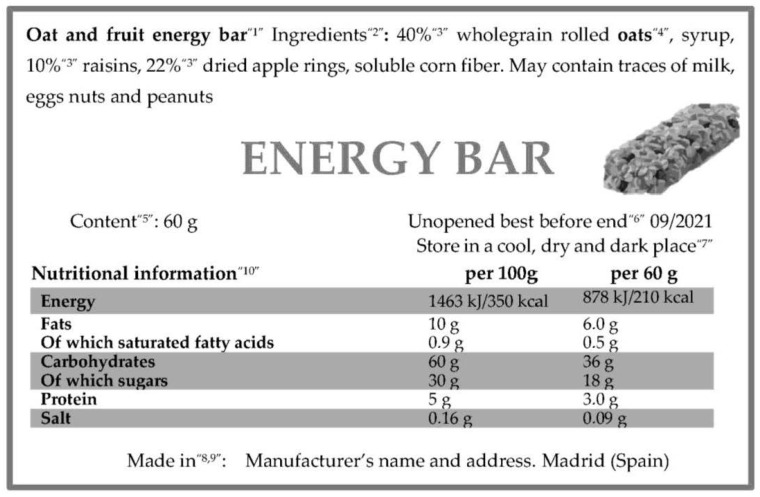
An example of a sport food label. Adapted from a commercial product. According to the European Regulation (Table 1), this sport food includes the following specific information. ^1^ The foodstuff designation (oat and fruit energy bar) that describes the product. ^2^ An ingredients list in descending order of weight, preceded by the word ingredients. ^3^ Quantity indication of certain ingredients or category of ingredients (oats, raisins, apple). The amounts of these ingredients must be indicated because they are mentioned in the name of the food (oat and fruit energy bar). ^4^ Substances causing allergies or intolerances (oats) clearly distinguished from the rest of the ingredients (in bold font in this case). ^5^ Net quantity (in grams). ^6^ Minimum durability date. ^7^ Storage conditions or conditions of use (store in a cool, dry and dark place). ^8^ Manufacturer’s name and address. ^9^ Country of origin or place of provenance. ^10^ Nutritional information including energy value and content of fat, saturated fat, carbohydrates, sugars, protein and salt. On this label, the mandatory information per 100 g is complemented with the nutritional information per portion or consumption unit (one bar).

In order to improve the understanding and use of food labelling, some examples labels for sport foods, both fortified and not, and supplements labels are included. Figure 1 shows, in an interpretive way, the label of a sport food, an energy bar, with all the mandatory mentions.

**Figure 2:**
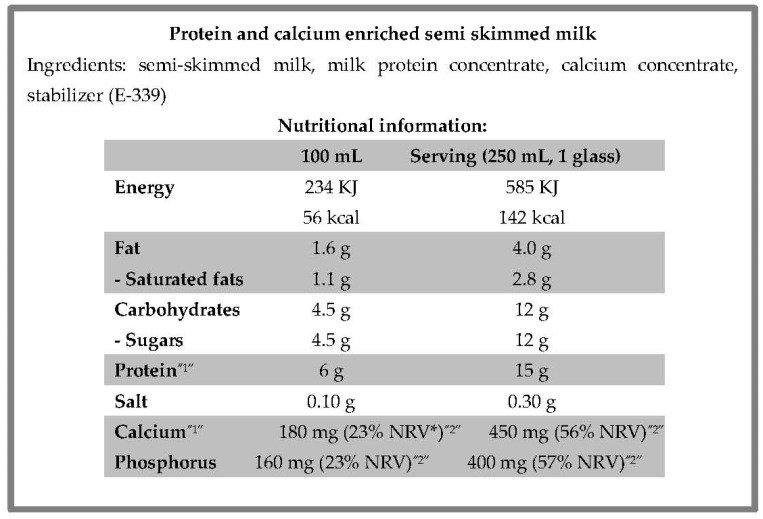
An example of the back of a fortified food label. Adapted from a commercial product. * nutrient reference values (NRVs). According to the European legislative framework, the mandatory nutrition declarations must be supplemented with an indication of the amounts of the added substances: ^1^ The amounts of protein (6 and 15 g per 100 g and serving) and calcium (180 mg and 450 mg per 100 g and serving). ^2^ In the case of vitamins and minerals, the percentage of the recommended daily amount of each one that is met per 100 g complemented with per serving (23% and 56% per 100 g or serving).

In addition to these mandatory requirements, if the sport food is fortified in a permitted substance, the labelling must refer to the added amounts of these substances. Figure 2 shows a practical example, with the information on vitamins and minerals expressed as a percentage of the daily reference intakes.

**Figure 3:**
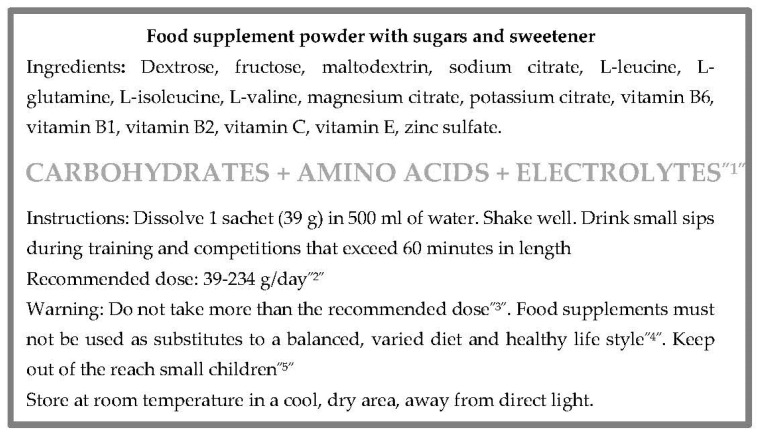
An example of the back of a supplement label. Adapted from a commercial product. In addition to some mandatory information, as explained above (ingredients, storage conditions and conditions of use), the back of the label refers to other particular information according to the European legislative framework about labelling of supplements [12]: ^1^ The names of the categories of nutrients or substances that characterize the product (carbohydrates + amino acids + electrolytes). ^2^ The portion of the product recommended for daily consumption (39–234 g/day). ^3^ A warning about not to exceed the stated recommended daily dose. ^4^ A statement about that food supplements should not be used as a substitute for a varied diet. ^5^ A warning about that this product should be stored out of the reach of young children.

Finally, a dietary supplement label is commented. Figure 3 shows the labelling of a dietary supplement with carbohydrates, amino acids and electrolytes. 

**Figure 4:**
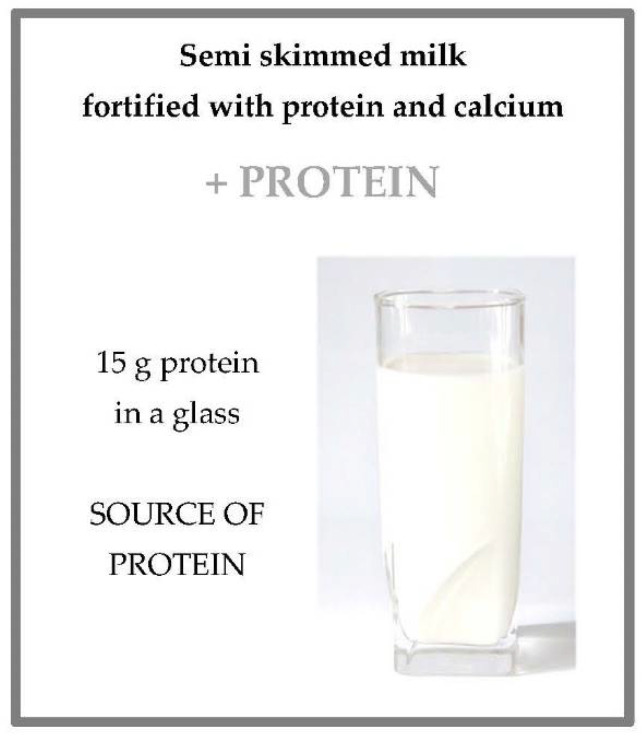
An example of the front of a fortified food label. Adapted from a commercial product.

**Figure 5:**
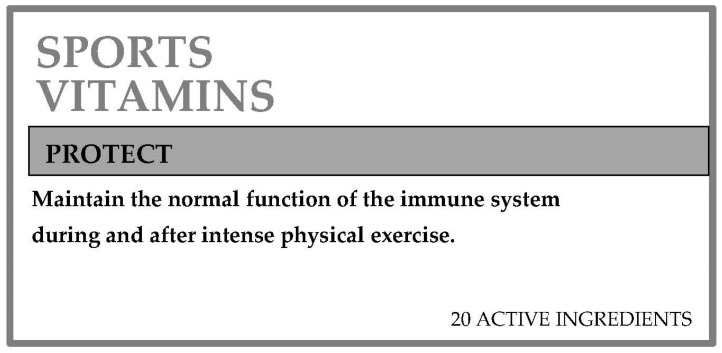
An example of the front of a dietary supplement label. Adapted from a commercial product.

To this end, two interpretive examples about the use of nutritional and health claims associated with a nutrient are included. Figure 4 shows the front of the label of a product with a nutritional claim associated with a nutrient in increased proportions. This front label refers to the amount of protein, shown in Figure 2 (protein and calcium enriched semi-skim milk). In the same way, Figure 5 shows an example of a label with an authorized health claim associated with a sport vitamin. 

According to the regulation mentioned above [20], this producer could inform consumers that this milk contains an increased proportion of protein compare to another milk by means of a nutritional claim (source of protein).

The health claim shown on this label is authorized for commercial use and is associated with vitamin C intake (Table 2).

## 5. Conclusions

Food labelling provides relevant information for making informed purchasing decisions. It has been recognized as a tool to protect consumers by ensuring food safety and nutritional quality.

Labels inform consumers about ingredients found in the product, specific nutrients and amounts, storage, cooking or preparation conditions, origin of the products, and the benefits associated with the product due to its nutrient content or contribution to a healthy diet. Also, labels are used to compare products though the purchases.

In the field of sport foods, all of the information that food labels contain is regulated by a legislative framework that may result in complex and confusing labeling by producers. It may be necessary to assist producers in understanding the information in order to avoid inappropriate labels in the marketplace. In the same terms, educational initiatives to help consumers make good use of labels may be necessary.

This type of manuscript could be a help to producers and consumers of sport foods and supplements because it explains and examines the principles that regulate sport food labelling. In addition, it could be of interest to sport nutrition professionals seeking to counsel appropriate food choices. In addition, readers can find some specific types of labels, such as sport food labels and supplement labels with specific mandatory information, fortified food labels with the mandatory nutritional information, and some practical examples of nutrition and health claims made by both sport foods and dietary supplements.

## Figures and Tables

**Table 1:** Mandatory information for packaged sport foods, fortified or not. This table includes particular requirements for each mention.

Mention	Particular Requirements
Foodstuff designation	If the product has a legal name, it must be used in the label. In its absence, the customary name or a descriptive name of the food can replace it. It may not be replaced by trademark, brand name or fancy name.
Ingredients list	The list of ingredients must be preceded by a heading with the word «ingredients». The list must include the ingredients in descending order of weight, including flavourings, food additives and food enzymes, and any constituent of a compound ingredient, used in the manufacture or preparation of a food and still present in the finished product.
Quantity indication of certain ingredients or category of ingredients	The indication of the quantity of an ingredient or category of ingredients used in the manufacture or preparation of a food shall be required where the ingredient or category of ingredients appears in the name of the food or is usually associated with that name by the consumer; or when this ingredient is emphasized on the labelling in words, pictures or graphics; or when is essential to characterize a food and to distinguish it from products with which it might be confused because of its name or appearance.
Substances causing allergies or intolerances	Must be indicated in the list of ingredients with a clear reference to the name of the substance or product emphasized through a typeset that clearly distinguishes it from the rest of the ingredients, for example by means of the font, style or background color. In the absence of a list of ingredients, the indication of the particulars referred shall comprise the word ‘contains’ followed by the name of the substance or products.
Net quantity	It must be expressed using liters, centiliters, milliliters, kilograms or grams. These expressions could be changed in order to ensure a better understanding by the consumer of the food information on the labelling.
Minimum durability date, ‘use by’ date and date of freezing	In the case of foods which, from a microbiological point of view, are highly perishable and are therefore likely after a short period to constitute an immediate danger to human health, the date of minimum durability shall be replaced by the ‘use by’ date.
Storage conditions or conditions of use	This information must be included when foods require special storage conditions and/or conditions of use. It could refer to appropriate storage or use of the food after opening the package, the storage conditions and/or time limit for consumption.
Manufacturer’s name and address	The company name and the address must be indicated.
Country of origin or place of provenance	Indication of the country of origin or place of provenance shall be mandatory to food business operators whenever its absence might to mislead consumers as to the true country of origin or place of provenance of that product, in particular if the information accompanying the food or the label as a whole would otherwise imply that the food has a different country of origin or place of provenance. Also, where the country of origin or the place of provenance of a food is given and it is not the same as that of its primary ingredient, the country of origin or place of provenance of the primary ingredient shall also be given; or (b) the country of origin or place of provenance of the primary ingredient shall be indicated as being different to that of the food.
Instructions for use	This information must be indicated to enable appropriate use to be made of the food, if it is necessary.
Nutritional information	The mandatory nutrition for sport foods must include the following: energy value and the amounts of fat, saturates, carbohydrate, sugars, protein and salt. Where appropriate, a statement indicating that the salt content is exclusively due to the presence of naturally occurring sodium may appear in close proximity to the nutrition declaration. The content of the mandatory nutrition declaration may be supplemented with an indication of the amounts of one or more of the following: monounsaturated fats; polyunsaturated fats; polyols; starch; fiber; any of the vitamins or minerals listed in the Regulation, present in significant amounts and expressed as a percentage of the maximum daily intakes set out in this Regulation. This information must be expressed per 100 g or per 100 mL. In addition, to the form of expression per 100 g or per 100 mL, the nutritional information may be expressed per portion or per consumption unit when it is easily recognizable by the consumer (for example per energy bar) or when portion size is referred in the label. In these cases, the information “per portion or per consumption unit” alone is permitted, however, the energy value shall be expressed per 100 g or 100 mL.

**Table 2:** Health claims related to physical exercise.

Nutrient/Substance	Declaration
Carbohydrates	Carbohydrates contribute to the recovery of normal muscle function (contraction) after highly intensive and/or long-lasting physical exercise leading to muscle fatigue and the depletion of glycogen stores in skeletal muscle.
Creatine	Daily consumption of creatine can reinforce the effect of resistance training on muscle strength in adults over 55 years of age.
	Creatine improves physical performance in successive sets of short, high-intensity exercise.
Vitamin C	Vitamin C contributes to maintain the normal function of the immune system during and after intense physical exercise.
Carbohydrate-based electrolyte solutions	The carbohydrate-based electrolyte solutions improve water absorption during exercise. It helps to maintain the resistance level in exercises that require prolonged resistance.
	The carbohydrate-based electrolyte solution improves water absorption during exercise.
